# Genetic influence on vascular smooth muscle cell apoptosis

**DOI:** 10.1038/s41419-024-06799-z

**Published:** 2024-06-08

**Authors:** David G. McVey, Catherine Andreadi, Peng Gong, Paulina J. Stanczyk, Charles U. Solomon, Lenka Turner, Liu Yan, Runji Chen, Junjun Cao, Christopher P. Nelson, John R. Thompson, Haojie Yu, Tom R. Webb, Nilesh J. Samani, Shu Ye

**Affiliations:** 1 Department of Cardiovascular Sciences and National Institute for Health Research Leicester Biomedical Research Centre, University of Leicester, Leicester, UK; 2 Cardiovascular-Metabolic Disease Translational Research Programme, Yong Loo Lin School of Medicine, National University of, Singapore, Singapore; 3 Shantou University Medical College, Shantou, China; 4 Department of Health Sciences, University of Leicester, Leicester, UK

**Keywords:** Apoptosis, Cell death

## Abstract

Vascular smooth muscle cell (VSMC) proliferation, migration, and apoptosis play important roles in many physiological processes and pathological conditions. To identify genetic influences on VSMC behavior, we measured these traits and undertook genome-wide association studies in primary umbilical artery-derived VSMCs from >2000 individuals. Although there were no genome-wide significant associations for VSMC proliferation or migration, genetic variants at two genomic loci (7p15.3 and 7q32.3) showed highly significant associations with VSMC apoptosis (*P* = 1.95 × 10^−13^ and *P* = 7.47 × 10^−9^, respectively). The lead variant at the 7p51.3 locus was associated with increased expression of the *GSDME* and *PALS2* genes in VSMCs. Knockdown of *GSDME* or *PALS2* in VSMCs attenuated apoptotic cell death. A protein co-immunoprecipitation assay indicated that GSDME complexed with PALS2. *PALS2* knockdown attenuated activated caspase-3 and *GSDME* fragmentation, whilst *GSDME* knockdown also reduced activated caspase-3. These findings provide new insights into the genetic regulation of VSMC apoptosis, with potential utility for therapeutic development.

## Introduction

Inter-individual variabilities in many biological and clinical traits in humans are well documented. It is plausible that there are also inter-individual variations at the cellular level, which warrants investigation. Genome-wide association studies (GWAS) of clinical phenotypes have been very successful in uncovering common genetic variants that confer disease predisposition and identifying candidate genes that are likely to play a role in disease pathogenesis [1]. Analogously, GWAS of cellular phenotypes, known as cellular GWAS, may unveil genetic variants that influence cellular characteristics and key players in the cognate biological processes [2–6]. For example, a previous study using this approach revealed the role of TUBB6 (tubulin, β 6 class V) and microtubule instability in inflammatory cell death [2].

Vascular smooth muscle cells (VSMCs) are a major component of the arterial wall and essential for arterial wall integrity. In healthy arteries, VSMCs proliferate only at a very low rate and their principal function is cell contraction to regulate vascular tone [7]. However, with the ability to change phenotype and behavior, VSMCs are a key player in many physiological processes and pathological conditions [7]. For instance, VSMC apoptosis contributes to the physiological closure of the infra-umbilical artery after birth and the closure of the ductus arteriosus following surgery [8]. VSMC migration, proliferation, and apoptosis participate in the pathogenesis of atherosclerosis that underlies coronary artery disease and several other vascular diseases, whilst VSMC apoptosis promotes atherosclerotic plaque rupture, arterial aneurysm development, and bypass vein grafting failure [8–12].

Apoptosis is known to involve a series of molecular and cellular events, with characteristic morphological changes such as nuclear fragmentation and nuclear condensation [13]. Apoptotic cells are taken up and eliminated by macrophages or other cells with phagocytic activity, a process known as efferocytosis [13]. In the absence of efferocytosis, apoptosis is generally followed by the breakdown of the plasma membrane, referred to as apoptotic cell death (ACD) or secondary necrosis, which represents a terminal phase of the apoptotic program characterized by plasma membrane permeabilization, cell swelling, and lysis, which is also characteristic of the pro-inflammatory pyroptotic cell death pathway [13].

In the present study, we observed substantial variations in proliferation, migration, and apoptosis among VSMCs from different individuals. To investigate possible genetic influences that might contribute to inter-individual variations in VSMC behavior, we performed cellular GWAS of cell proliferation, migration, and apoptosis using a large VSMC biobank. The study detected 2 genomic loci, respectively, on chromosome 7p15.3 and 7q32.3, that were associated with VSMC apoptosis. Fine-mapping analyses and further functional experiments indicated that the association of the 7p15.3 locus with VSMC ACD was mediated by a genetic effect on the expression of the *GSDME* (Gasdermin E) and *PALS2* (Protein Associated with LIN7 2, MAGUK P55 family member) genes and that both GSDME and PALS2 promote VSMC ACD.

## Materials and methods

### VSMC biobank

The VSMC biobank consisted of VSMCs isolated from the artery of umbilical cords from 2114 donors and stored at passage 3 in either freezing medium under liquid nitrogen or RNAlater solution (Sigma-Aldrich) for nucleic acid isolation at −20 °C, as previously described [14]. As shown previously, the cells expressed high levels of the VSMC marker α-smooth muscle actin but not the fibroblast marker TE7 and did not express the endothelial cell marker von Willebrand factor [14].

The umbilical cord tissues used in this study were collected and provided by the Anthony Nolan Trust with ethical approval and informed consent of the donor’s parents.

### Genotyping and imputation

DNA extracted from samples (*n* = 2016) of the VSMC biobank was genotyped for 760,000 variants with the use of the Illumina Global Screening Array Multi-Diseases v2.0 bead-chips, followed by imputation to obtain genotypic information for 7,334,165 variants, as detailed previously [14].

### VSMC behavior assays

VSMCs at passage 3 were subjected to proliferation, migration, and apoptosis assays, as detailed previously [14] and outlined below.

In the proliferation assay, cells (from 2025 donors) were incubated with 10 µM EdU for 6 h and then fixed with 3.7% formaldehyde for 15 min, followed by measurement of EdU incorporation with the use of the BaseClick EdU HTS 488 kit (Sigma-Aldrich, BCK-HTS488-20) and an Operetta CLS High-Content Analysis System (PerkinElmer). Images were analyzed with Harmony 4.8 software (PerkinElmer).

In the migration assay, cells (from 2,019 donors) were stained with 10 µM CellTracker Green CMFDA (5-chloromethylfluorescein diacetate) (Thermo Fisher, C7025) and 0.4 µM Hoechst 33342 (AnaSpec, AS-83218), and then imaged every 1 h for 16 h using the Operetta CLS High-Content Analysis System. Cells tracked for the full 16 h were used to calculate the straightness, accumulated distance, and displacement parameters with Harmony 4.8 software, and all cells with 2 or more consecutive time points were used to calculate the migration speed.

In the apoptosis assay, cells (from 2075 donors) were stained with Hoechst 33342 (1.6 µM) (AnaSpec, AS-83218) and propidium iodide (0.5 µM) (Sigma-Aldrich, P4864), and imaged on the Operetta CLS High-Content Analysis System. Then, the cells were removed from the instrument and the media replaced with media containing Hoechst 33342, propidium iodide and the apoptosis inducer staurosporine (2.5 µM) (Alfa Aesar, J62837). Thereafter, the cells were returned to the Operetta CLS and incubated for 30 min before being imaged every 30 min for 16 h. The images were analyzed with Harmony 4.8 software.

In each of the above assays, each VSMC preparation was analyzed in 4 replicate wells on a 96-well plate. As previously described [14], all three assays had a low coefficient of variation: 2.99% (95% confidence interval 2.12–3.87%) for the proliferation assay, 1.81% (1.50–2.12%) for the migration assay, and 3.01% (1.62–4.53%) for the apoptosis assay. To verify that the cell behavior data generated were reproducible, we previously performed repeated experiments at two different times (one at passage 3 and the other at passage 4) on each of a random selection of the VSMC preparations (*n* = 50) [14]. The results of the two independent assays were highly consistent [14], indicating high reproducibility of the data generated.

### Cellular GWAS

Prior to cellular GWAS, KING kinship analysis [15] was performed, using PLINK [16, 17], to identify relatedness among the samples in the dataset using the array variants. Sample pairs with a KING kinship coefficient >0.0442 (indicating a third-degree or closer relationship) were identified and one sample from each pair was removed at random. The following covariates were included in the cellular GWAS: the top 5 principal components (PCs), sex, use of cells for apoptosis/ACD assay following cryopreservation, the use of kanamycin antibiotic during culture at each passage, and the month of isolation of cells from tissue. The imputed SNP genotype data were filtered to remove SNPs with low MAF (<0.01), SNPs with HWE *p* value <1 × 10^−6^ and SNP missingness <0.05. The resulting SNP dataset was then used for GWAS analysis using rank-based inverse normally transformed data of VSMC behavior parameters and the aforementioned covariates using PLINK [16, 17]. The VSMC behavior parameters analyzed were proliferation (EdU Total, EdU High, and EdU Low), migration (straightness, speed, distance, and displacement), and apoptosis/ACD (NA30m, NA60m, NF30m, NF60m, %DC4h, %DC8h, and TT50D). SNPs with a *P* value <5 × 10^−8^ in the additive genetic model were classified as reaching genome-wide significance. Manhattan plots were prepared using a Python script (https://github.com/pgxcentre/manhattan_generator).

### ATAC-sequencing

ATAC-sequencing was performed on 3 VSMC samples using a protocol described by Buenrostro et al. [18], with minor modifications. In brief, 5 × 10^4^ VSMCs were lysed with cold lysis buffer (10 mM Tris-HCl, pH7.4, 10 mM NaCl, 3 mM MgCl_2_, 0.1% NP-40, 0.1% Tween-20, and 0.01% Digitonin), and then the nuclei were incubated with a transposition reaction buffer containing Tn5 transposases (Illumina, FC-121-1030) for 30 min at 37 °C. The resulting libraries were subjected to a polymerase chain reaction (PCR) with high-fidelity polymerase (New England Labs, M0541) and Nextera barcodes (primers listed in Supplementary Table S5). After purification using a PCR cleanup kit (Qiagen), the quality and quantity of the libraries were assessed with the use of a Bioanalyzer system (Agilent) and a Qubit 4 fluorometer (Thermo Fisher). The libraries were then multiplexed and sequenced using the Novaseq 6000 SP Flow Cell (Illumina) with >100 million reads (50 bp pair-end) per individual indexed library.

ATAC-sequencing data were processed using Galaxy (https://usegalaxy.eu and https://usegalaxy.org) [19, 20]. In brief, the quality of the raw data was evaluated with FastQC (v0.11.9). Adapters were removed using trimmomatic (v0.38), and paired-end reads aligned to the human genome (hg38) using Bowtie2 (v2.4.2) with the --very-sensitive, --dovetail and --X 1000 maximum insert size parameters. Mitochondrial and duplicate reads were removed using bamtools (v2.4.0) and Picard MarkDuplicates (v2.18.2), resulting in an average of 45 million filtered reads per sample for downstream analyses. Peaks were called using MACS2 (v2.1.1.20160309) with the start sites of the reads extending by 200 bp (100 bp in each direction) to assess coverage and a *P* value 0.05 cutoff to reveal open chromatin peaks. The intersect function of BedTools v2.30.0 was used to identify overlaps of MACS2 peaks with the chromosome 7p15.3 and 7q23.3 loci at which variants were found to be associated with VSMC apoptosis/ACD in this study. Pygenometracks (v3.6) was used for visualization of genomic data tracks with publicly available ENCODE ChIP-seq SMC embryo-origin datasets (ENCSR116JEF: H3K27ac experiment ENCSR210ZPC, H3K4me3 experiment ENCSR515PKY and H3K27me3 experiment ENCSR143RMH) and the generation of MACS2 peak and coverage files.

### RNA-sequencing

Total RNA was extracted from an aliquot of passage 3 VSMCs stored in RNAlater solution, with the use of the Biobasic EZ-10 DNAaway RNA miniprep kit (Biobasic). RNA-sequencing was performed on RNA samples with a concentration >70 ng/µl, an RNA integrity number (RIN) ≥6.8, and 260:280 and 260:230 ratios of ≥2. A strand-specific library with rRNA removal was prepared from the total RNA of each of the 1499 VSMC samples, and 150 bp paired-end sequencing at a 30 million read depth was performed with the Illumina platform. The RNA-sequencing data were processed using the following software pipeline: FastQC v0.11.5 [21] for quality check, BBMap v38.51 [22] for adapter trimming, STAR v2.7.1a [23] for alignment, SAMtools v1.9 [24] for processing aligned reads, Rsubread for read quantification, and MultiQC v1.3 [25] for summarizing the output of other tools. The read count was normalized with DESeq2 [26].

### eQTL and sQTL analyses

eQTL and sQTL analyses were performed on 1486 VSMC samples that had both genotype and RNA-sequencing data. SNPs with missingness >1%, minor allele frequency <1%, or Hardy–Weinberg equilibrium test *P* value <1 × 10^−6^ in the eQTL sample subset were removed with PLINK 2.0 [17]. PLINK was also used to calculate the PCs and recode the genotype data in 0,1,2 format based on the dosage of the alternative allele. The first 15 PEER (probabilistic estimation of expression residuals) factors for the gene expression data were calculated with the R package PEER [27, 28]. The eQTL analysis was performed using linear additive regression to model the effect of each SNP located within 1 Mb of the transcription start site of each gene with the R package matrixEQTL [29]. Sex, the first two PCs and the first ten PEER factors were used as covariates. The eigenMT-BH method [30–32] was used for multiple testing correction. The sQTL analysis was performed with MatrixEQTL [29] using leafCutter normalized splice-clusters ratio. Sex, the first two genotype PCs and the first 50 PCs of the splice-clusters ratio were included as covariates in the linear model.

### eCAVIAR analysis

An eCAVIAR analysis [33] was performed on cellular GWAS summary statistics and eQTL data from this VSMC biobank study. In this analysis, LD was estimated using the VSMC genotype data, and the number of causal SNPs was set to 2. We used colocalization posterior probability (CLPP) ≥0.05 as the threshold for significant colocalization. The R package LocusCompareR (v.1.0) [34] was used to visualize the colocalization signals.

### SMR analysis

An SMR analysis [35] was also performed with the cellular GWAS summary statistics and eQTL data from this VSMC biobank study. This was carried out using the SMR (v1.03) software tool (https://cnsgenomics.com/software/smr/) with its default setting and the significance threshold *P* < 0.05 for SMR. LD was estimated using the VSMC genotype data.

### siRNA-mediated gene knockdown

Cells randomly selected from the VSMC biobank were transfected with either a *GSDME* siRNA (Thermo Fisher, s230618) or a scramble siRNA (Thermo Fisher, 4390843), with Lipofectamine RNAiMAX Transfection Reagent (Thermo Fisher, 13778150). Efficient knockdown of GSDME was validated by Western blotting of protein extracts from transfected cells and an anti-GSDME antibody (Thermo Fisher, MA5-36092).

Similarly, cells randomly selected from the VSMC biobank were transfected with either a *PALS2* siRNA (Thermo Fisher, s28519) or a scramble siRNA (Thermo Fisher, 4390843). Efficient knockdown of PALS2 was validated by Western blotting of protein extracts from transfected cells and an anti-PALS2 antibody (Thermo Fisher, PA5-82663).

### Western blotting analysis

Protein extracts were prepared from VSMCs with the use of RIPA (radioimmunoprecipitation assay) lysis buffer (50 mM Tris-HCl pH8, 150 mM NaCl, 2 mM EDTA pH8, 1% NP-40, 0.5% sodium deoxycholate, and 0.1% sodium dodecyl sulfate). Protein extracts were subjected to Western blotting analysis with the following antibodies, respectively: an antibody recognizing amino acids 34–214 of GSDME (Thermo Fisher, MA5-36092), an antibody recognizing amino acids 317–466 of GSDME (Sigma-Aldrich, HPA011326), an anti-PALS2 antibody (Thermo Fisher, PA5-82663), an antibody for cleaved caspase-3 (Asp175) (Cell Signaling, #9661), an antibody for full-length and cleaved caspase-7 (Cell Signaling, #9492), an anti-ACTB antibody (Sigma-Aldrich, A1978), or an antibody against GAPDH (glyceraldehyde 3-phosphate dehydrogenase) (Proteintech, 60004-1). The Western blotting experiments were replicated at least twice.

### Co-immunoprecipitation

VSMC lysates (total protein extracts) were prepared with the use of RIPA lysis buffer supplemented with a protease inhibitor cocktail (Sigma-Aldrich, 05056489001). Protein concentrations were quantified with the use of a BCA protein assay kit (Thermo Fisher, J63283.QA). Immunoprecipitation (IP) was performed using either an anti-GSDME antibody (Thermo Fisher, MA5-36092) or an anti-PALS2 antibody (Thermo Fisher, PA5-21889), with cross-linking of antibodies to Dynabeads G (Thermo Fisher, 10003D). An IgG isotype control antibody (Thermo Fisher, 10500 C) was used as the negative control for IP. The immunoprecipitants were subjected to sodium dodecyl sulfate–polyacrylamide gel electrophoresis, with a solution equivalent to 5% of the amount of cell lysate used for IP, as a loading control. Subsequently, Western blotting analysis was performed using an anti-GSDME antibody (Thermo Fisher, MA5-36092) and an anti-PALS2 antibody (Thermo Fisher, PA5-21889), respectively. In an additional experiment, IP from VSMC lysates were performed with the use of a different anti-GSDME antibody (Abcam, ab225893, lane 2) or an IgG isotype control antibody (Thermo Fisher, 10500 C, lane 3), without cross-linking of antibodies to Dynabeads, followed by Western blotting with the anti-GSDME antibody, an anti-PALS2 antibody (Thermo Fisher, PA5-82663), or an anti-caspase-3 antibody (Cell Signaling, #9662).

The membrane-enriched fraction from VSMCs was prepared with the use of a Mem-PER Plus Membrane Protein Extraction Kit (Thermo Fisher, 89842), with a protease inhibitor cocktail (Sigma-Aldrich, 05056489001) added to the permeabilization and solubilization buffers of the kit. Protein concentrations were quantified with the use of a BCA protein assay kit (Thermo Fisher J63283.QA). IP was performed using an anti-GSDME antibody (Thermo Fisher, MA5-36092) and Dynabeads G (Thermo Fisher, 10003D), or using an anti-PALS2 antibody (Thermo Fisher, PA5-21889) and a 1:1 mix of Dynabeads A and Dynabeads G (Thermo Fisher, 10003D). IgG1 (Sigma-Aldrich, M5284) and IgG (Thermo Fisher, 10500 C) were used as the negative control for IP. The immunoprecipitants were subjected to sodium dodecyl sulfate–polyacrylamide gel electrophoresis, with a solution equivalent to 5% of the amount of membrane fraction sample used for IP, as loading controls. Subsequently, Western blotting analysis was performed using antibodies for GSDME (Thermo Fisher, MA5-36092) and PALS2 (Thermo Fisher, PA5-21889), respectively.

### Phosphoproteomics analysis

VSMCs (Thermo Fisher, C0175C) transfected with either *PALS2* siRNA (*n* = 4) or scramble siRNA (*n* = 4) were subjected to a phosphoproteomics analysis. Acetone-precipitated protein samples were dissolved in S-TRAP buffer (50 mM triethylammonium bicarbonate and 5% sodium dodecyl sulfate), and further processed using the S-Trap micro column (Protifi) according to the manufacturer’s recommendations. A total of 100 μg of digested peptides from each sample was labeled with 8-plex iTRAQ reagents (SCIEX) according to the manufacturer’s recommendations. iTRAQ-labeled peptides from each sample were pooled and subjected to phosphopeptide enrichment using the High-Select Fe-NTA Phosphopeptide Enrichment Kit (Thermo Fisher Scientific), according to the manufacturer’s protocol. The phosphopeptide-enriched sample was analyzed by one-dimension online reversed-phase nanoLC (liquid chromatography) coupled to mass spectrometry with the use of a TripleTOF 6600 instrument (SCIEX).

The liquid chromatography-mass spectrometry data were processed with the use of ProteinPilot 5.02 Software (SCIEX) and the Swissprot Human reference proteome (UP000005640, 2022_04 release, 20397 entries) spiked with common contaminant proteins (cRAP), using the thorough search mode with the Phospho Emphasis special factor and a protein detection score threshold of 0.05. A decoy database search was performed to estimate the false discovery rate (FDR) for phosphopeptide identification. A 1% global FDR was used as the threshold to generate the list of identified phosphopeptides.

A Gene Ontology Enrichment analysis (http://geneontology.org/) was performed on phosphorylated proteins showing a change of ≥2 folds with *P* < 0.05 in VSMCs transfected with *PALS2* siRNA compared with VSMCs transfected with scramble siRNA. A pathway enrichment bubble plot was generated with the use of SRplot (http://www.bioinformatics.com.cn/srplot).

### Statistical analyses

#### Power calculation

The power was calculated in terms of the percentage of the variance explained by a genetic variant using the genpwr package in R assuming an additive model that is correctly specified. The calculations were performed for a sample size of 1800 and a significance level of 5 × 10^−8^.

Cellular GWAS was performed using an additive genetic model as described earlier in the Cellular GWAS section. eQTLs and sQTLs were tested using a linear additive regression model as described in the eQTL and sQTL section. The two-tailed Wilcoxon test was used to ascertain a difference in ACD between cells transfected with *GSDME* siRNA or *PALS2* siRNA and cells transfected with scramble siRNA (negative control).

## Results

### Among-individual variation in VSMC behavior

We performed proliferation, migration, and apoptosis assays on primary cultures of VSMCs from different individuals (*n* > 2000), using a high-throughput, high-content analysis system. The assays were carried out on cells at passage 3, with four technical replicates for each sample. Proliferation was assayed by measuring the incorporation of EdU (5-ethynyl-2’-deoxyuridine) into nascent DNA in proliferating cells with the use of a fluorescence-based detection method, from which we obtained data for the total percentage of EdU-positive cells (hereafter referred to as EdU Total), the percentage of EdU-positive cells with fluorescence intensity >10,000 (EdU High), and the percentage of EdU-positive cells with fluorescence intensity <10,000 (EdU Low). In the migration assay, we obtained data on migration straightness, speed, distance, and displacement. In the apoptosis assay, we incubated cells with the apoptosis inducer staurosporine and measured the following parameters: the decrease in the nuclear area at 30 and 60 min (referred to as NA30m and NA60m, respectively) post-staurosporine treatment, nuclear fragmentation index at 30 and 60 min (NF30m and NF60m), the percentage of cells that were propidium iodide positive and therefore considered dead cells at 4 and 8 h (%DC4h and %DC8h), and the length of time for 50% of cells to become propidium iodide positive (TT50D).

**Fig. 1:**
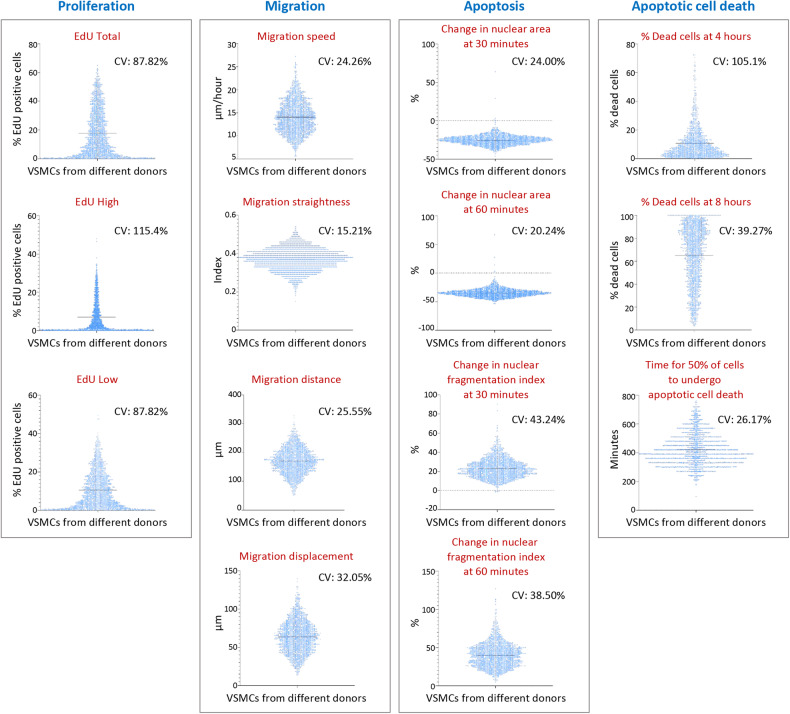
Variations of behavior of vascular smooth muscle cells from different individuals. Scatter plots showing variations of parameters of vascular smooth muscle cell proliferation, migration, apoptosis, and apoptotic cell death. In each plot, the blue dots represent vascular smooth muscle cell samples from different individuals and the horizontal line indicates the mean value. VSMCs vascular smooth muscle cells, CV coefficient of variation; nuclear fragmentation index: coefficient of variation of the pixel intensity in the nucleus; cell death: positive propidium iodide staining.

The above assays showed considerable variations in proliferation, migration, and apoptosis, among VSMCs from different individuals, with the coefficients of variation ranging from 20.24 to 115.4% (Fig. 1). As previously described [14], results from repeated assays of a random selection of samples from this VSMC biobank displayed high correlations, indicating that the inter-individual variability in VSMC behavior is a biological characteristic rather than a technical artifact.

### Genetic associations with VSMC apoptosis

Having observed considerable inter-individual variability in VSMC behavior, we undertook cellular GWAS to ascertain a possible genetic influence. A power calculation indicated that the study had 80% power to detect a genetic variant that explained ~2.2% of the variance in VSMC behavior (Supplementary Fig. S1). No genetic association was detected with VSMC proliferation or migration at the genome-wide significance threshold (*P* < 5 × 10^−8^). However, genetic variants at two genomic loci (7p15.3 and 7q32.3) showed highly significant associations with VSMC apoptosis.

**Fig. 2:**
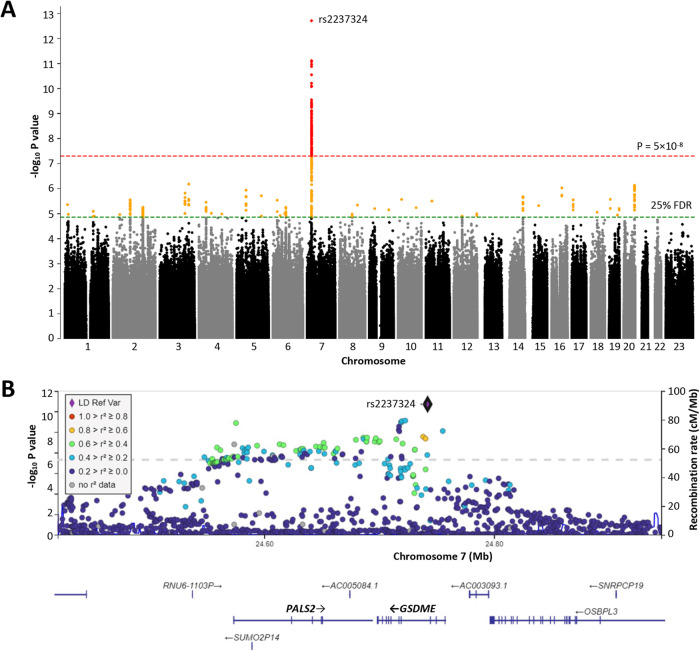
Association of genetic variants at the chromosome 7p15.3 locus and apoptotic cell death. **A** Manhattan plot showing an association between variants (indicated by red dots) on chromosome 7p15.3 and the percentage of apoptotic dead cells in cultured vascular smooth muscle cells following treatment with the apoptosis inducer staurosporine for 4 h (%DC4h), at the genome-wide significance level (*P* < 5 × 10^−8^, indicated by the red horizontal dotted line). **B** Regional plot of the 7p15.3 locus in relation to %DC4h. The y-axis indicates −log_10_ *P* value for associations of the 7p15.3 variants (represented by colored dots) with %DC4h. Different colors of the dots indicate different degrees of linkage disequilibrium of the different genetic variants with the index SNP rs2237324. The locations of the *PALS2* and *GSDME* genes are shown at the bottom of the plot.

At the chromosome 7p15.3 locus, a cluster of 91 single nucleotide polymorphisms (SNPs) in linkage disequilibrium (LD) were associated with VSMC ACD (Fig. 2 and Supplementary Table S1). Among these 91 SNPs, rs2237324 showed the most significant associations with VSMC ACD (*P* = 1.95 × 10^−13^) (Fig. 2 and Supplementary Table S1).

At the chromosome 7q32.3 locus, a cluster of 9 SNPs in LD were associated with nuclear fragmentation of VSMCs (Supplementary Fig. S2 and Supplementary Table S2), with rs62468397 showing the strongest association with this NF60m (*P* = 7.47 × 10^−9^) (Supplementary Fig. S2 and Supplementary Table S2).

### The lead variant at the chromosome 7p15.3 locus is located in an open chromatin region

**Fig. 3:**
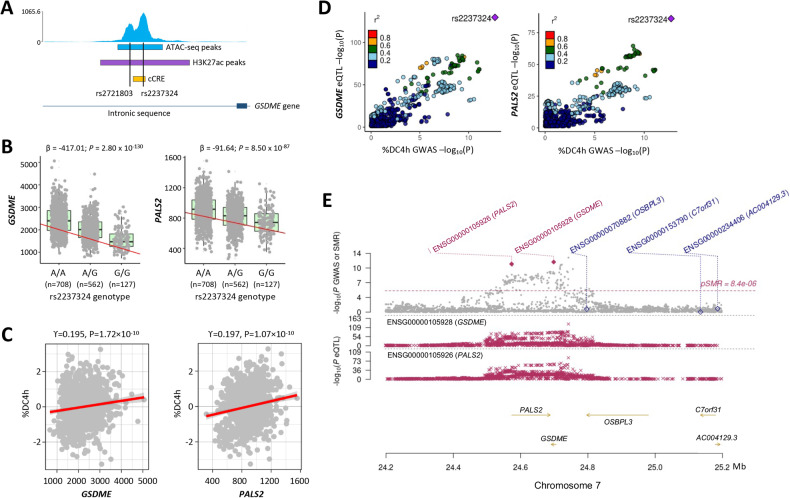
Causal genes at the chromosome 7p15.3 locus associated with apoptotic cell death. **A** The lead SNP rs2237324 at the 7p15.3 locus overlaps with peaks of ATAC-sequencing of vascular smooth muscle cells, peaks of H3K27Ac, and a candidate *cis*-regulatory element (cCRE) (ENCODE Accession: EH38E2540815). **B** Scatter plots of expression values of *GSDME* (left panel) and *PALS2* (right panel) by genotype of the lead SNP rs2237324 at the 7p15.3 locus. Gray dots represent different vascular smooth muscle cell samples. β- and *P* values are from linear additive regression analysis. **C** Scatter plots of correlations of %DC4h (y-axis) with the expression values (x-axis) of GSDME (left panel) and PALS2 (right panel). Gray dots represent different vascular smooth muscle cell samples. Correlation coefficient (ϒ)- and *P* values are from Pearson correlation analysis. **D** LocusZoom plots showing colocalization of %DC4h cellular GWAS signal with *GSDME* eQTL signal (left panel) and *PALS2* eQTL signal (right panel). The dots represent different single nucleotide polymorphisms (SNPs) and are colored according to their linkage disequilibrium values (*r*^2^). **E** Chromosome 7p15.3 regional plot showing %DC4h associations and eQTLs. Gray dots in the upper plot represent the *P* values for SNPs from the cellular GWAS of %DC4h, whilst diamonds represent the *P* values for genes from the summary-data-based Mendelian randomization (SMR) test. Filled diamonds highlight genes that were significant in the SMR test (*P* < 0.05). The genes that were significant in the SMR test are listed in red text, whilst the genes that were non-significant in the test are listed in blue text. The red crosses in the lower plots show the *P* values of eQTLs of *GSDME* and *PALS2*.

ATAC-sequencing (Assay for Transposase Accessible Chromatin using Sequencing) of VSMC samples (*n* = 3) showed that the lead SNP rs2237324 at the chromosome 7p15.3 locus resides in an open chromatin region. Additionally, a bioinformatic analysis using the UCSC Genome Brower revealed that rs2237324 was located in a candidate *cis*-regulatory element (cCRE) with enhancer-like signatures including high max-Z scores of DNase I hypersensitivity, H3K27Ac, H3K4me3, and/or CCCTC-binding factor, in various tissues including artery and smooth muscle cells (Fig. 3A).

### ACD-associated variant at the chromosome 7p15.3 locus influences *GSDME* and *PALS2* expression

**Fig. 4:**
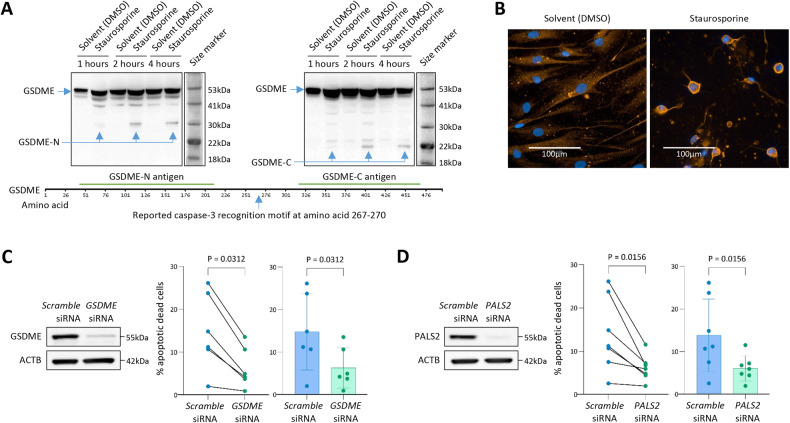
Attenuation of GSDME or PALS2 reduces apoptotic cell death. **A** Western blot images showing the appearance of the GSDME N-fragment (labeled GSDME-N) and C-fragment (labeled GSDME-C) from vascular smooth muscle cells (VSMCs) incubated with either staurosporine (2.5 µM) or solvent (dimethylsulfoxide, DMSO) for 1, 2, and 4 h, respectively. **B** Images of GSDME immunostaining of VSMCs treated with either staurosporine (2.5 µM) or solvent (DMSO) for 8 h. Orange color indicates GSDME positive staining. **C** Left: Western blot images showing efficient knockdown of GSDME in VSMCs transfected with *GSDME* siRNA. Middle and Right: Percentages of apoptotic dead VSMCs at 4-h post-staurosporine treatment (mean ± standard deviation is shown in the column chart on the right), *n* = 6 donors, *P* values from two-tailed Wilcoxon test. **D** Left: Western blot images showing efficient knockdown of PALS2 in VSMCs transfected with PALS2 siRNA. Middle and Right: Percentages of apoptotic dead VSMCs at 4-h post-staurosporine treatment (mean ± standard deviation is shown in the column chart on the right), *n* = 7 donors, *P* values from a two-tailed Wilcoxon test.

The chromosome 7p15.3 region contains several protein-coding genes (*GSDME*, *PALS2*, and *OSBPL3*), two long non-coding RNA genes (*AC005084.1* and *AC003093.1*) and several pseudogenes (*RNU6-11.3* *P, SNRPCP19*, and *SUMO2P14*) (Fig. 2). An eQTL (expression Quantitative Trait Loci) analysis of transcriptomics data from RNA-sequencing of cells (*n* = 1499 donors) from the VSMC biobank showed that the ACD-associated variants at the 7p15.3 locus exerted an eQTL effect on the expression of the *GSDME* and *PALS2* genes. The ACD-promoting allele (the A allele) of the lead SNP rs2237324 was associated with higher expression of both *GSDME* (β = 417.01; *P* = 2.80 × 10^−130^) and *PALS2* (β = 91.64; *P* = 8.50 × 10^−87^) (Fig. 3B). Accordantly, a correlation was observed between increased ACD and higher expression levels of *GSDME* and *PALS2* (Fig. 3C). An sQTL (splicing Quantitative Trait Loci) analysis detected no significant effects from the ACD-associated variants on gene splicing. Based on data from the GTEx Portal (https://gtexportal.org/home/), the RNA expression levels of *GSDME* and *PALS2* in arteries (where VSMCs are a major cell type) are comparable to their expression levels in many other tissues, although some other tissues (such as lymphocytes and the certain brain tissues) express higher levels of these two genes (Supplementary Fig. S3). According to the Human Protein Atlas (https://www.proteinatlas.org/), VSMCs have a high level of GSDME protein but no detectable PALS2 protein (Fig. S4). Our study shows that both *GSDME* and *PALS2* are expressed in VSMCs (Fig. 3B) and that there are substantial between-individual differences in the expression levels of these two genes (Fig. 3B), an important finding from our study of VSMCs from a very large group of individuals (*n* = ~1500). Such between-individual differences in the expression levels of these genes are not reflected in the currently available databases, as these databases do not have information on the expression levels of these genes in VSMCs from a very large number of individuals as in our study. Additionally, our study shows that both the GSDME and PALS2 proteins are present in VSMCs (Fig. 4A and Supplementary Fig. S5).

To identify the causal gene(s) at the 7p15.3 locus, we performed an eCAVIAR (eQTL and GWAS CAusal Variants Identification in Associated Regions) analysis [33] on cellular GWAS and eQTL data from the VSMC samples. The analysis revealed that at the 7p15.3 locus, the signal of genetic association with ACD colocalized with the signal of eQTL effect on the expression of *GSDME* and *PALS2* (Supplementary Table S3), suggesting that both *GSDME* and *PALS2* were potential causal genes. Figure 3D shows a visual representation of the colocalization.

To further determine the causal gene(s) at the 7p15.3 locus, we performed a Mendelian randomization analysis. Results of a summary-data-based Mendelian randomization (SMR) test [35] on cellular GWAS and eQTL data from the VSMC samples further indicated that both *GSDME* and *PALS2* were potential causal genes (Fig. 3E and Supplementary Table S4).

The second apoptosis-associated locus detected in this study, i.e., 7q32.2, contained two protein-coding genes (*PLXNA4* and *CHCHD3*), several long non-coding RNA genes (AC105443.1, AC009365.1 AC018643.1, AC011625.1, FLJ40288, and AC009365.2), and a pseudogene (CAPZA1P4) (Supplementary Fig. S2). However, the apoptosis-associated variants at this locus did not show any discernable eQTL or sQTL effect, suggesting that the association between this locus and apoptosis was unlikely to be due to an influence on gene expression or splicing.

### Knockdown of GSDME or PALS2 reduces VSMC ACD

A Western blotting analysis detected a 30 kDa N-fragment and a 25 kDa C-fragment, of GSDME, in protein extracts from VSMCs treated with staurosporine to induce apoptosis, whilst only the full-length GSDME (55 kDa) was present in protein extracts from untreated VSMCs (Fig. 4A). In line with the finding from recent studies that the GSDME-N fragment forms pores in the plasma membrane of dying apoptotic cells [36], we observed GSDME enrichment on the surface of apoptotic VSMCs in an immunocytochemical analysis (Fig. 4B).

To test if the expression level of the *GSDME* gene could influence VSMC ACD, we carried out siRNA-mediated knockdown of GSDME in VSMCs and then performed an apoptosis assay. The experiment showed that GSDME attenuation resulted in a substantial decrease in VSMC ACD (Fig. 4C).

To investigate if PALS2 could also affect apoptosis, we performed an apoptosis assay in VSMCs with siRNA-mediated knockdown of PALS2. The assay showed that PALS2 attenuation also reduced VSMC ACD (Fig. 4D).

### GSDME complexes with PALS2 complexes

**Fig. 5:**
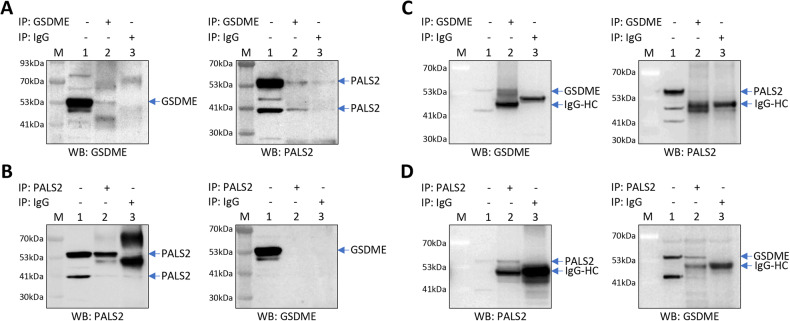
GSDME complexes with PALS2. **A**, **B** Lysates of vascular smooth muscle cells were subjected to immunoprecipitation (IP) with the use of either an anti-GSDME antibody (Thermo Fisher, MA5-36092) (lane 2 in **A**), an anti-PALS2 antibody (Thermo Fisher, PA5-21889) (lane 2 in **B**), or an IgG isotype control antibody (Thermo Fisher, 10500 C) (lane 3 in **A**, **B**), with cross-linking of antibodies to Dynabeads to reduce the IgG heavy chain band, followed by Western blotting with the anti-GSDME antibody or the anti-PALS2 antibody, as indicated in the figure. **C**, **D** Membrane fraction of vascular smooth muscle cells were subjected to IP with the use of either the anti-GSDME antibody (lane 2 in **C**), the anti-PALS2 antibody (lane 2 in **D**), or the IgG isotype control antibody (lane 3 in **C**, **D**), followed by WB with the anti-GSDME antibody or the anti-PALS2 antibody, as indicated in the figure. IgG-HC IgG heavy chain. **A**–**D** Lane 1:5% of the amount of the protein extract used for immunoprecipitation. M: protein size markers.

Since PALS2 is capable of interacting with other proteins [37], we wondered if PALS2 could complex with GSDME. To investigate this, we performed co-immunoprecipitation (co-IP) experiments. A co-IP assay of VSMC lysates observed PALS2 in immunoprecipitant prepared using an anti-GSDME antibody (Fig. 5A and Supplementary Fig. S6) but did not find GSDME in immunoprecipitant prepared using an anti-PALS2 antibody (Fig. 5B). However, a co-IP assay of VSMC membrane fraction samples detected PALS2 in immunoprecipitant prepared using an anti-GSDME antibody (Fig. 5C), as well as GSDME in immunoprecipitant prepared using an anti-PALS2 antibody (Fig. 5D), suggesting that GSDME and PALS2 could form a complex.

### Influence of PALS2 on caspase-3/7 and other proteins

**Fig. 6:**
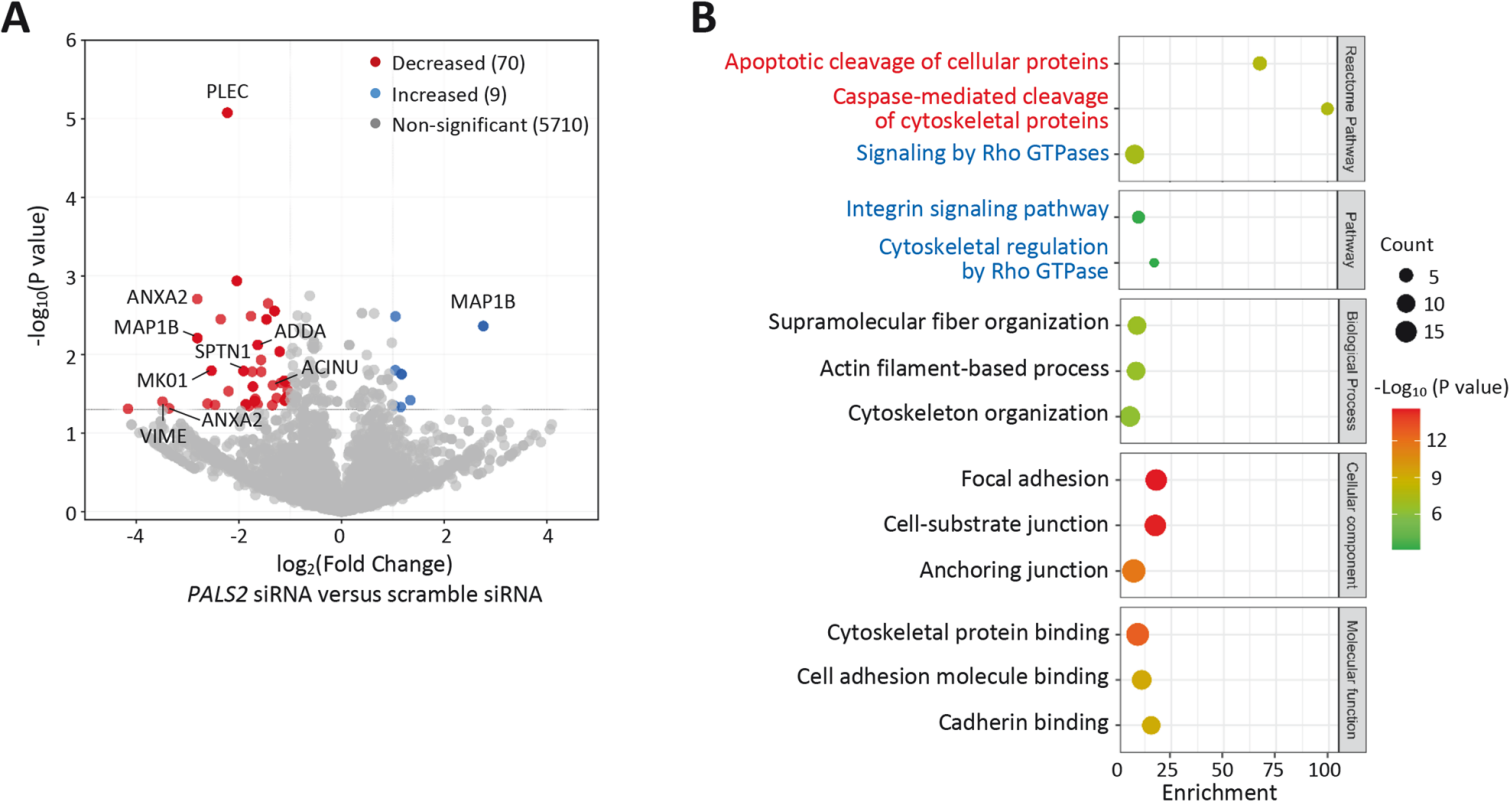
Results of phosphoproteomics analysis of vascular smooth muscle cells with PALS2 knockdown. **A** Proteins with increased or decreased phosphorylation in cells transfected with *PALS2* siRNA compared with cells transfected with scramble siRNA. **B** Key outputs from gene ontology enrichment analyses of proteins with a change ≥2 folds (Log_2_ fold ≥1 or ≤−1) and *P* < 0.05 (−log_10_ *P* value <1.3) in the phosphoproteomics assay.

Since PALS2 has the ability to interact with other proteins [37], we performed a phosphoproteomics analysis comparing VSMCs with and without PALS2 knockdown, to investigate if PALS2 could influence the phosphorylation status of other proteins. The analysis identified 39 proteins whose phosphorylation status changed in VSMCs with PALS2 knockdown (Fig. 6A and Supplementary Tables S6, S7). A gene ontology analysis of these proteins showed enrichment of several pathways, including pathways involved in the apoptotic cleavage of cellular proteins, and caspase-mediated cleavage of cytoskeletal proteins (Fig. 6B). The proteins in these pathways, influenced by PALS2 knockdown, were ACINU (apoptotic chromatin condensation inducer in the nucleus), ADDA (alpha-adducin), PLEC (Plectin), SPTN1 (spectrin alpha chain, non-erythrocytic 1), and VIME (vimentin), whose functions are summarized in Supplementary Tables S8, S9.

**Fig. 7:**
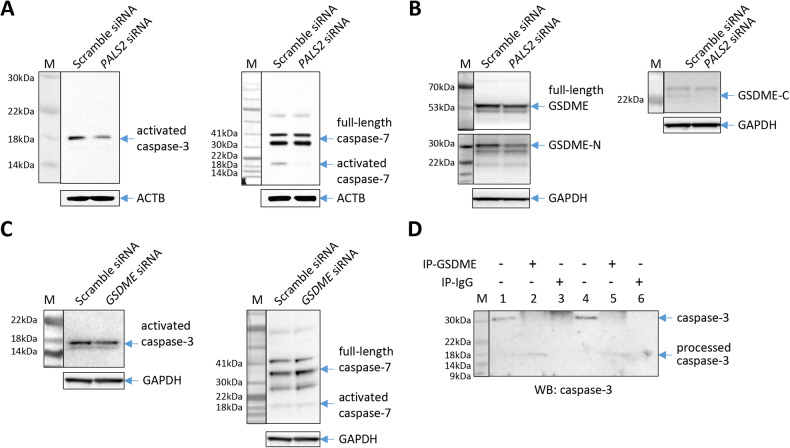
PALS2 knockdown attenuates GSDME fragmentation and caspase-3/7 activation, whilst GSDME knockdown reduces caspase-3 activation. Western blot images showing less activated caspase-3 and less activated caspase-7 in PALS2 siRNA-transfected vascular smooth muscle cells compared with scramble siRNA-transfected vascular smooth muscle cells (**A**), less GSDME N-terminal fragment and C-terminal fragment in PALS2 siRNA-transfected vascular smooth muscle cells compared with scramble siRNA-transfected vascular smooth muscle cells (**B**), and a reduction of activated caspase-3 but not activated caspase-7 in GSDME siRNA-transfected vascular smooth muscle cells compared with scramble siRNA-transfected vascular smooth muscle cells (**C**), with staurosporine treatment. **D** Results of a co-immunoprecipitation assay showing caspase-3 in immunoprecipitants prepared using an anti-GSDME antibody in vascular smooth muscle cells transfected with either PALS2 siRNA (lanes 4, 5, and 6) or scramble siRNA (lanes 1, 2, and 3). Lanes 1 and 4: 0.5% of the amount of the protein extract used for immunoprecipitation. **A**–**D** M: protein size markers.

In agreement, Western blotting analyses showed that VSMCs with PALS2 knockdown had lower levels of cleaved/activated caspase-3 and cleaved/activated capase-7, than VSMCs without PALS2 knockdown (Fig. 7A).

Furthermore, Western blotting analyses demonstrated that PALS2 knockdown resulted in a reduction in the amounts of GSDME N-fragment and C-fragment, indicating that PALS2 depletion attenuates GSDME fragmentation (Fig. 7B).

### Influence of GSDME on caspase-3

Interestingly, our experiment showed that knockdown of GSDME also reduced cleaved/activated caspase-3 in VSMCs but did not affect the amount of cleaved/activated caspase-7 (Fig. 7C). A co-IP experiment indicated that GSDME complexed with caspase-3 (Fig. 7D), and their interaction was not affected by PALS2 knockdown (Fig. 7D), suggesting that the influence of PALS2 on caspase-3 activation was not a result of an effect of PALS2 on the interaction between GSDME and caspase-3.

## Discussion

**Fig. 8:**
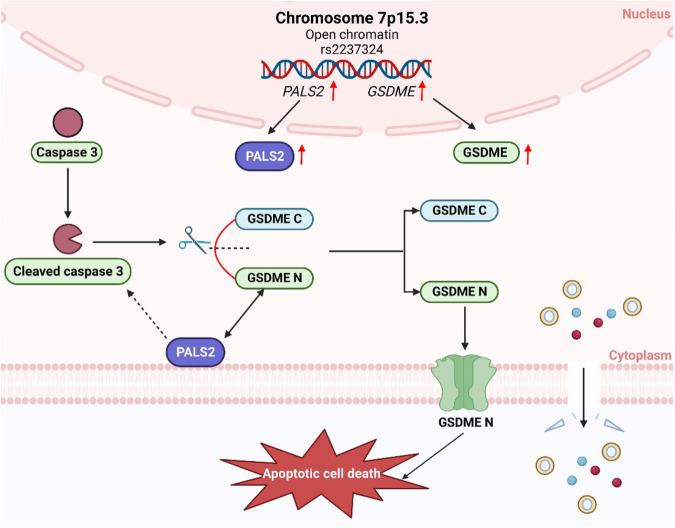
Summary of findings from this study. Our study reveals that genetic variation at the chromosome 7p15.3 locus increases the expression of the *GSDME* and *PALS2* genes and that higher levels of *GSDME* and *PALS2* promote apoptotic cell death. Additionally, this study indicates that PALS2 can be complex with GSDME and increase the level of activated caspase-3. Previously, studies by other groups have shown that cleavage of GSDME by caspase-3 releases the GSDME N-fragment, which in turn is incorporated into the plasma membrane to form pores and cause cell death.

Our study, based on the largest number of VSMC samples studied to date, provides novel insights into the genetic regulation of VSMC behavior. Although we did not detect any genome-wide significant associations for VSMC proliferation or migration, genetic variants at two genomic loci (7p15.3 and 7q32.3) showed highly significant associations with VSMC apoptosis. Furthermore, our functional studies identified *GSDME* and *PALS2* as the likely causal genes mediating the genetic association at the 7p15.3 locus (Fig. 8). These findings are relevant for a better understanding of the regulation of VSMC ACD, an event occurring in many physiological processes and pathological conditions [8–12].

Recent studies have revealed that GSDME plays a key role in the progression of apoptosis to ACD [36, 38]. In this process, caspase-3 cleaves GSDME to release its N-terminal moiety, which in turn binds to the plasma membrane and forms pores therein, thus triggering ACD [36, 38, 39]. Studies have shown that GSDME-mediated ACD occurs only in GSDME-positive cells (cells that express GSDME, e.g., human umbilical artery smooth muscle cells) but not in GSDME-negative cells (e.g., human umbilical vein endothelial cells) [36, 38]. One of the novel findings of our study is that the expression level of *GSDME* in GSDME-positive cells has an influence on ACD and that *GSDME* expression is under the influence of local genetic polymorphism(s). In line with findings from other studies that GSDME can promote caspase-3 activation to form a self-amplifying feed-forward loop [39, 40], our study showed that GSDME complexed with caspase-3 and that GSDME knockdown reduced the amount of activated caspase-3 in VSMCs.

PALS2 (also known as MAGUK P55 Subfamily Member 6, and membrane palmitoylated protein 6, MPP6) belongs to the membrane-associated guanylate kinases (MAGUK) family. Several members of the MAGUK family have been reported to form complexes with transmembrane, cytoskeletal, or cytoplasmic signaling proteins, and thereby regulate cell signaling, cell behavior, and intercellular junctions [41]. Previous studies have shown that PALS2 can interact with CADM1 (cell adhesion molecular 1, also known as NECL2), CADM3, CASK (calcium/calmodulin-dependent serine protein kinase), LIN7 (protein LIN7), SRC (SRC proto-oncogene, non-receptor tyrosine kinase), and CNTNAP2 (contactin associated protein 2, also known as CASPR2), respectively [37, 42]. Additional novel findings of our study are that PALS2 can complex with GSDME and also contributes to the regulation of cleaved/activated caspase-3 and cleaved/activated caspase-7 levels. Furthermore, our study shows that PALS2 knockdown reduces GSDME fragmentation and VSMC ACD. In line with these findings, our study additionally reveals that PALS2 influences the phosphorylation status of a variety of proteins, several of which are players in pathways involved in the apoptotic cleavage of cellular proteins, and caspase-mediated cleavage of cytoskeletal proteins, suggesting that PALS2 can promote caspase activation and ACD via these pathways.

In addition to the findings regarding the chromosome 7p15.3 locus, our study observed an association between variants on chromosome 7q32.3 and VSMC apoptosis characterized by increased nuclear fragmentation. However, no eQTL effects were detected from variants at this locus. The mechanism underlying this genetic association is currently unclear.

As mentioned earlier, VSMC apoptosis plays an important role in the development of atherosclerosis and several other pathological conditions [8–12, 43]. Interestingly, studies have shown that VSMCs are potent and efficient phagocytes of apoptotic VSMCs [44, 45]. However, in atherosclerotic lesions, phagocytosis of apoptotic cells by VSMCs and macrophages is severely impaired, resulting in secondary necrosis and the release of pro-inflammatory cytokines that promote atherosclerosis progression [45–47]. The results of our study imply that GSDME and PALS2 may have a role in this process, which warrants investigation in future studies.

An interrogation of the PhenoScanner database (http://www.phenoscanner.medschl.cam.ac.uk/) [48] showed a nominal association between the chromosome 7p15.3 locus and coronary artery disease (CAD). Specifically, the VSMC ACD-promoting allele (A allele) of the index SNP rs2237324 at this locus is nominally associated with increased CAD susceptibility [β = 0.02, P = 2.65 × 10^−2^, *n* = 332,477 in a CAD GWAS using the UK Biobank [49], and β = 0.02, *P* = 2.56 × 10^−2^, *n* = 184,305 in a GWAS meta-analysis of CAD [50]]. Additionally, the PhenoScanner interrogation showed a nominal association of SNP rs62468397 at the chromosome 7q32.3 locus with cardiovascular disease risk, with its anti-apoptosis (C allele) being nominally associated with lower risk of acute ischemic heart disease (β = −0.005, *P* = 3.17 × 10^−2^, in samples of the UK Biobank, *n* = 337,199) and lower risk of death caused by rupture of thoracic aortic aneurysm (β = −0.004, *P* = 8.03 × 10^−3^, in samples of the UK Biobank, *n* = 7637).

There is evidence indicating that chemotherapy drugs kill certain normal human cells through GSDME-mediated pyroptotic cell death, which contributes to the toxicity and side effects of chemotherapy [38]. The finding of our study that genetic variants at the 7p15.3 locus influence *GSDME* expression and ACD raises the question as to whether 7p15.3 genotyping can be utilized to help identify individuals at high risk of developing chemotherapy side effects. Examination of this hypothesis may provide opportunities to provide improved chemotherapy with reduced side effects without reducing treatment efficacy.

In this study, no genetic association with VSMC proliferation or migration was detected. However, this cannot preclude the possibility of associations existing between these cellular traits and genetic variants that have a small effect size. As described earlier, our study utilizing the biobank of VSMCs from >2000 individuals had 80% power to detect a genetic variant that explained approximately 2.2% of the variance in VSMC behavior. Although this biobank is by far the largest VSMC collection that has been reported, it is plausible that an even larger biobank is required for detecting genetic influence on cell behavior from variants that have a small effect size.

In summary, our study shows that there is an inter-individual variation in VSMC behavior and that VSMC ACD is under a genetic influence. The results of this study indicate that the association between the chromosome 7p15.3 locus and VSMC ACD is mediated by a genetic influence on the expression of *GSDME* and *PALS2*. Furthermore, our study reveals a previously unknown biological role of PALS2, namely, it plays a role in VSMC ACD. These findings provide a new insight into the genetic influence at the cellular level and the regulation of VSMC apoptosis, with potential utility for therapeutic development and personalized medicine.

### Supplementary information

Supplementary Figures

Supplementary Tables

Uncropped original Western blot images

## Data Availability

The datasets generated and/or analyzed during the current study are available from the corresponding author on reasonable request.
