# Supplementary material for: Genetic influence on vascular smooth muscle cell apoptosis
Source: Cell Death Dis. 2024 Jun 8;15(6):402. doi: 10.1038/s41419-024-06799-z (PMC11162461; doi:10.1038/s41419-024-06799-z)
Supplement: Supplementary file 1 — Supplementary Figures [file 41419_2024_6799_MOESM1_ESM.pdf]

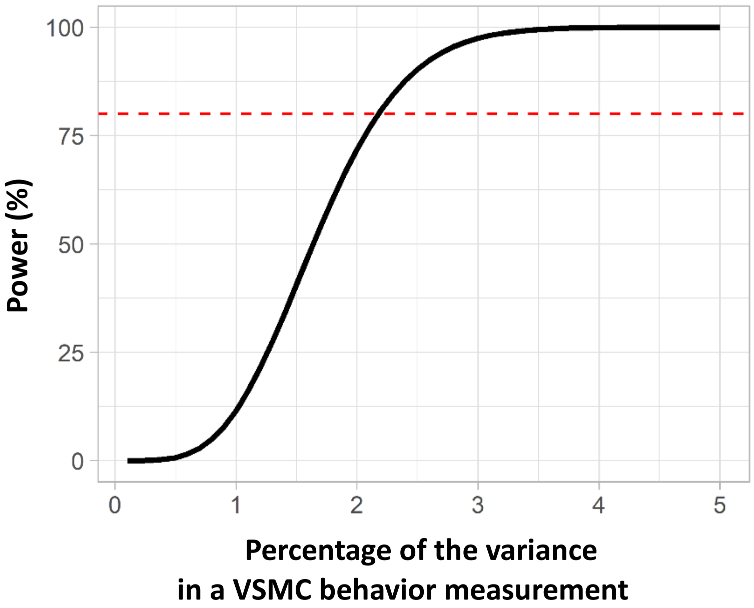

Supplementary Figure S1. Power curve

Power to detect a genetic variant under an additive model for a sample size of 1,800 and a significance level of $P < 5\times10^{-8}$.

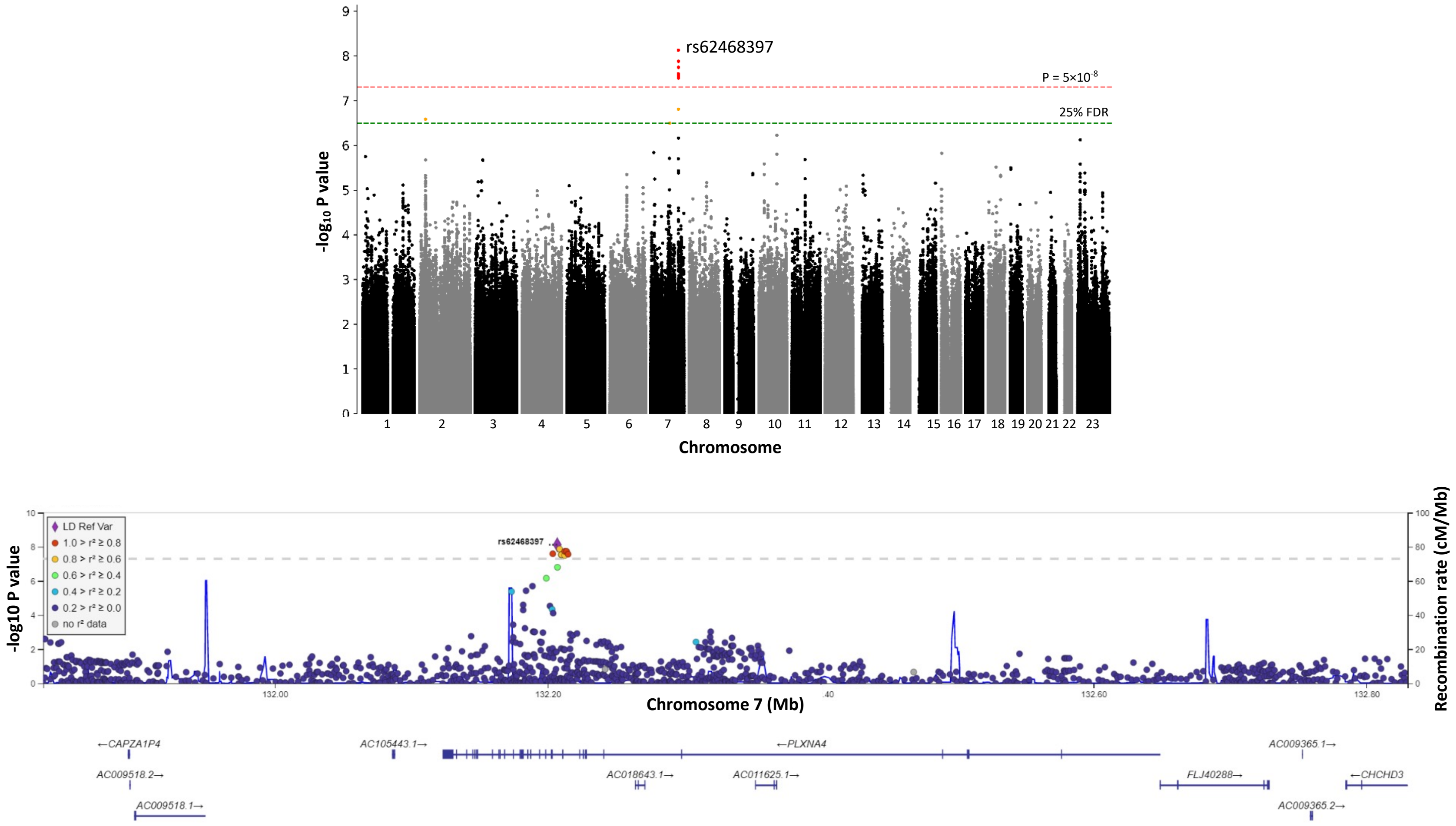

Supplementary Figure S2. Association between chromosome 7q32.3 genetic variants and apoptosis

A. Manhattan plot showing an association between variants (indicated by red dots) on chromosome 7q32.3 and nuclear fragmentation index in cultured VSMCs following treatment with the apoptosis inducer staurosporine for 60 minutes (NF60m), at the genome-wide significance level ($P < 5\times10^{-8}$, indicated by the red horizontal dotted line). **B**. Regional plot of the 7q32.3 locus in relation to NF60m. The y-axis indicates $-\log_{10}$ P-value for associations of the 7q32.3 variants (represented by colored dots) with NF60m. Different colors of the dots indicate different degrees of linkage disequilibrium (LD) of the different genetic variants with the index SNP rs62468397.

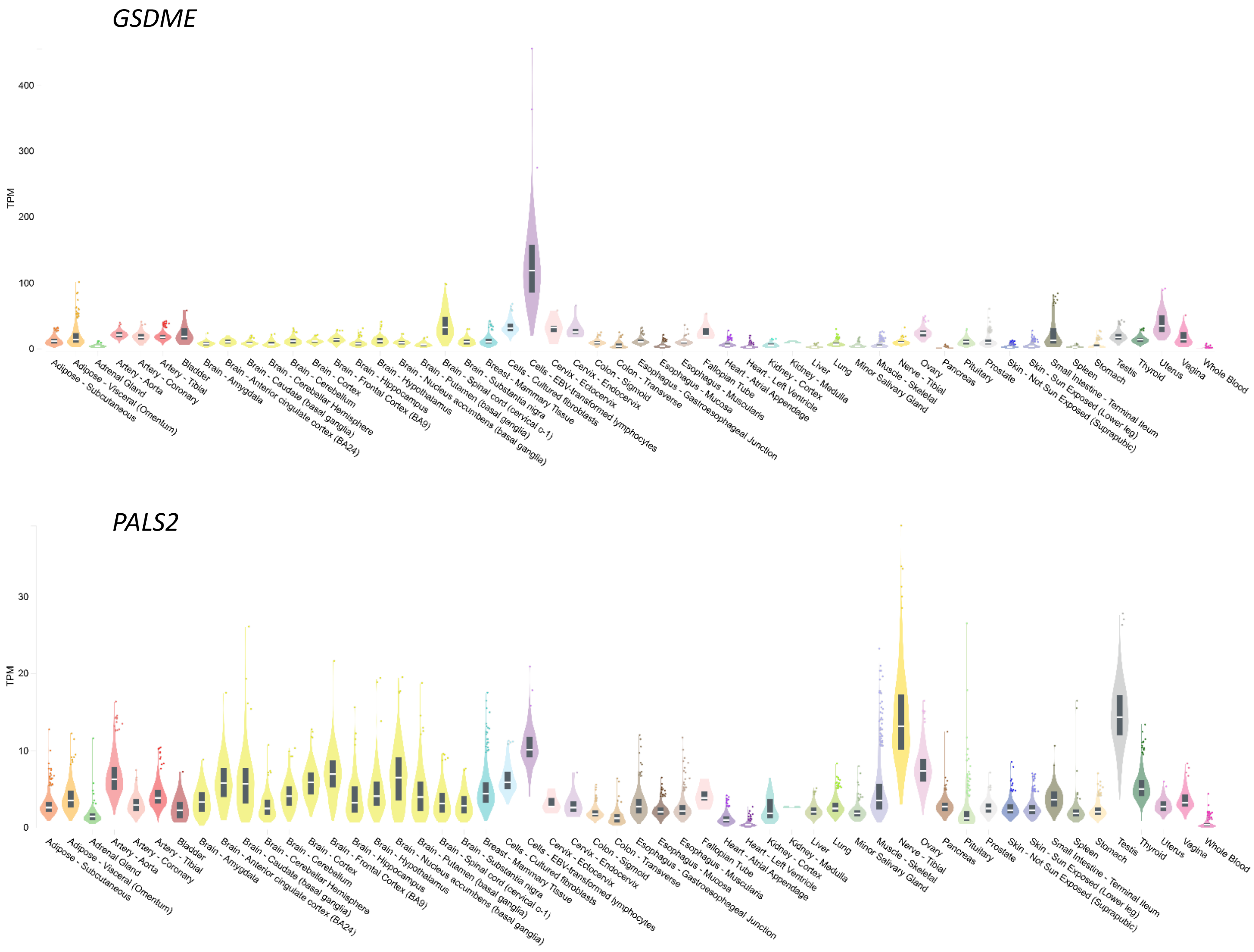

Supplementary Figure S3. Expression levels of *GSDME* and *PALS2* in various tissues

Data from GTEx Portal (https://gtexportal.org/home/)

GSDME

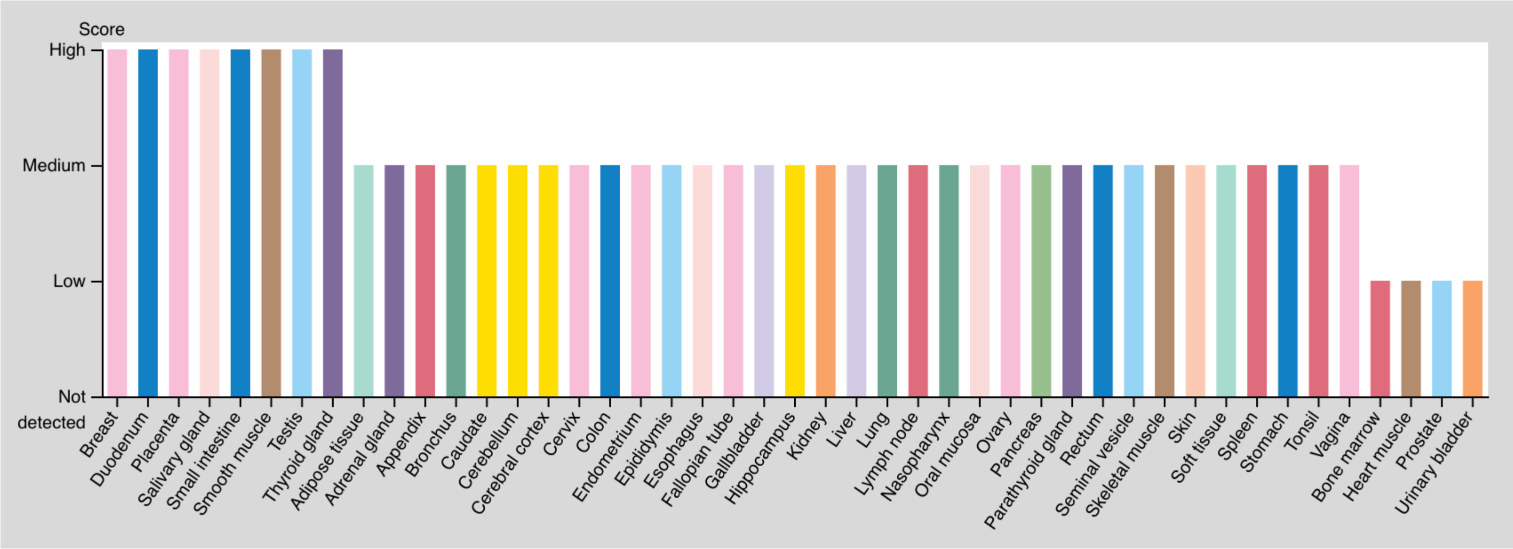

PALS2

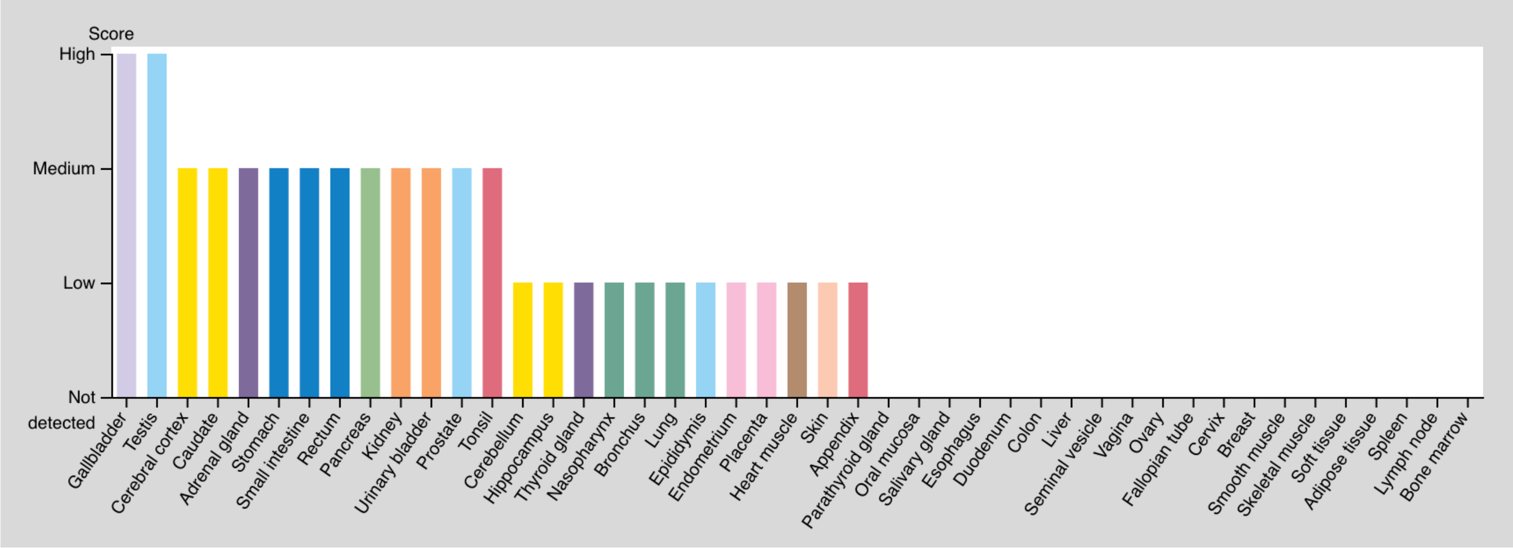

Supplementary Figure S4. Expression levels of *GSDME* and *PALS2* in various tissues
Data from the Human Protein Atlas (https://www.proteinatlas.org/)

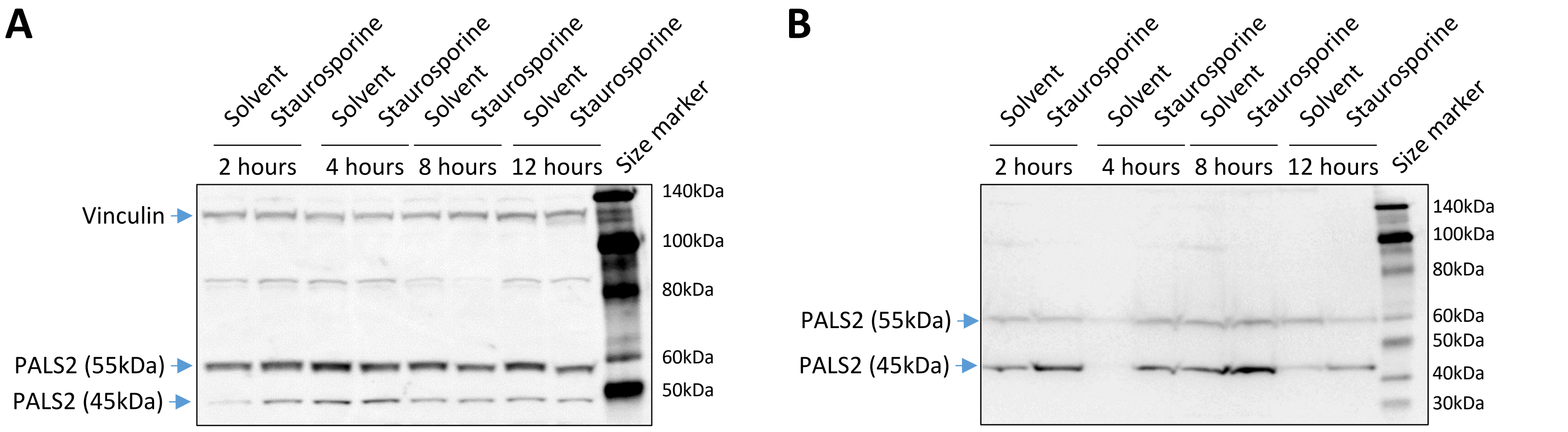

Supplementary Figure S5. PALS2 Protein in vascular smooth muscle cells

Western blotting of whole protein extracts (**A**) and membrane fractions (**B**) of vascular smooth muscle cells (VSMCs). Vinculin serves as a loading control.

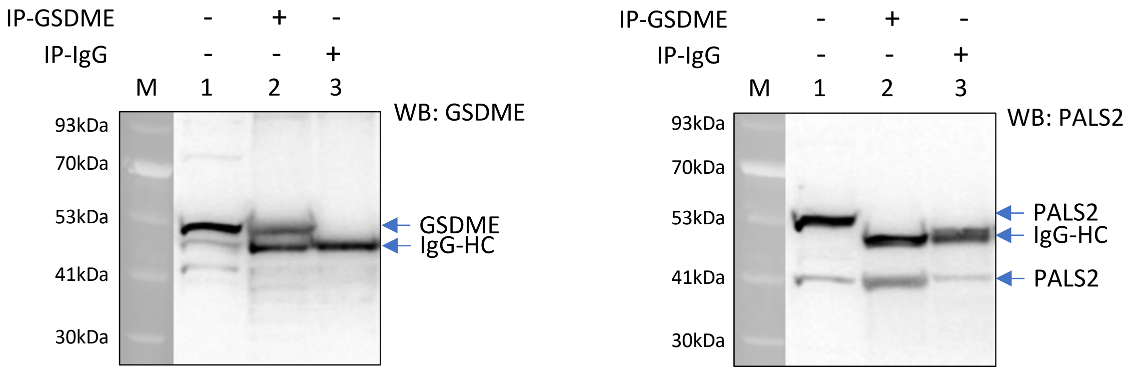

Supplementary Figure S6. GSDME complexes with PALS2

Vascular smooth muscle cell lysates were subjected to immunoprecipitation with the use of either an anti-GSDME antibody (Abcam, ab225893, lane 2) or an IgG isotype control antibody (ThermoFisher, 10500C, lane 3), followed by Western blotting with the anti-GSDME antibody (left) or an anti-PALS2 antibody (ThermoFisher, PA5-82663)(right). Lane 1: 5% of the amount of the protein extract used for immunoprecipitation. M: protein size markers. IgG-HC: IgG heavy chain.
