# Supplementary material for: Genetic influence on vascular smooth muscle cell apoptosis
Source: Cell Death Dis. 2024 Jun 8;15(6):402. doi: 10.1038/s41419-024-06799-z (PMC11162461; doi:10.1038/s41419-024-06799-z)

Uncropped original Western blot image for Figure 4A (left)

(The area shown in Figure 4A is indicated by the blue line)

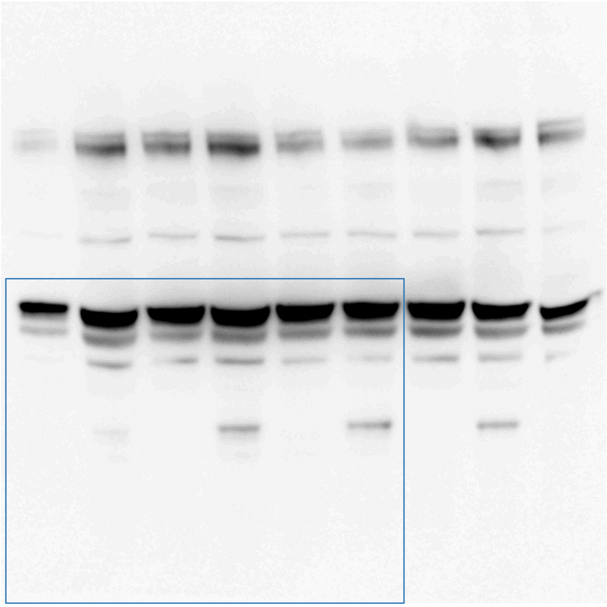

Uncropped original Western blot image for Figure 4A (right)

(The area shown in Figure 4A is indicated by the blue line)

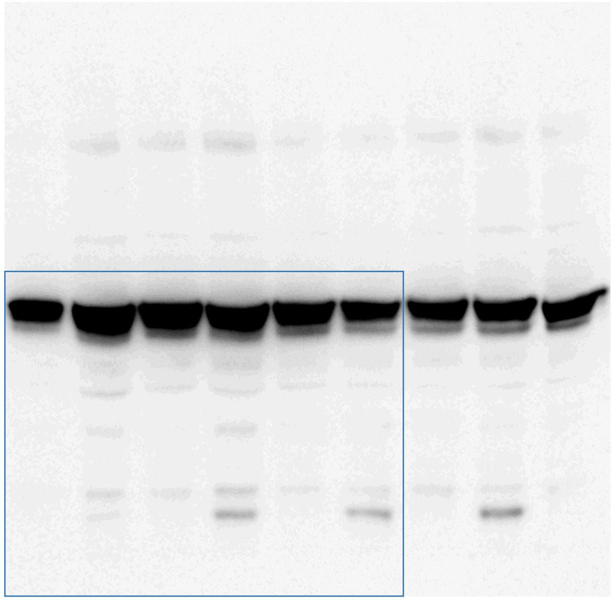

Uncropped original Western blot image for Figure 4C & 4D

(The areas shown in the Figure are indicated by the blue lines)

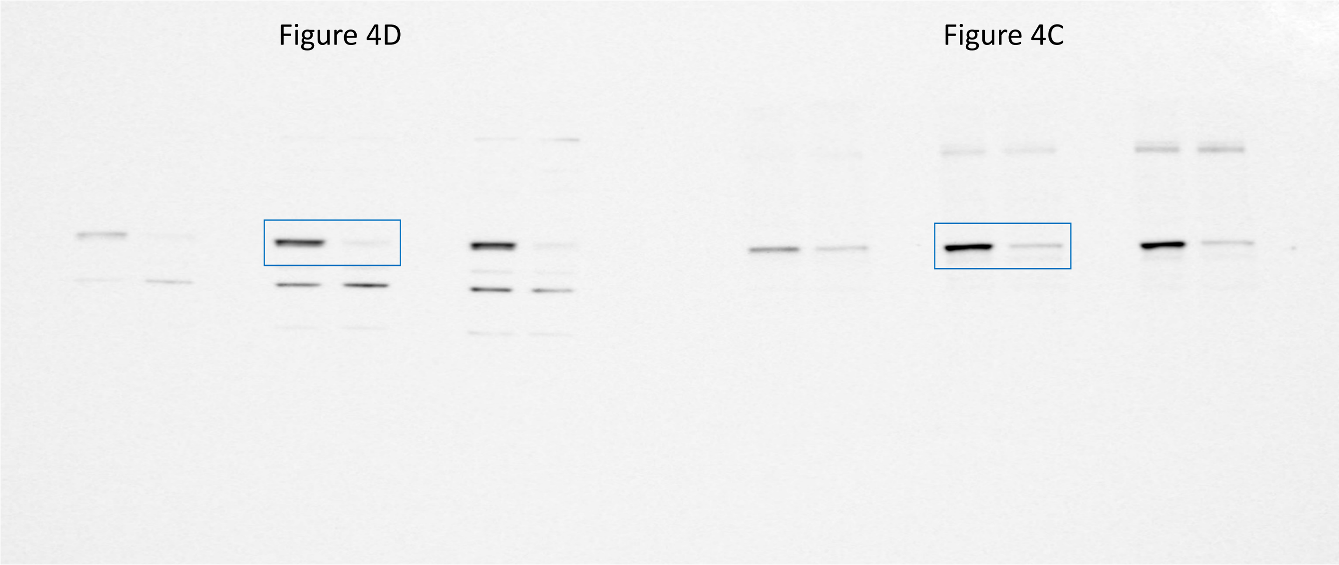

Uncropped original Western blot image for Figure 5A (left)
(The area shown in Figure 5A (left) is indicated by the blue line)

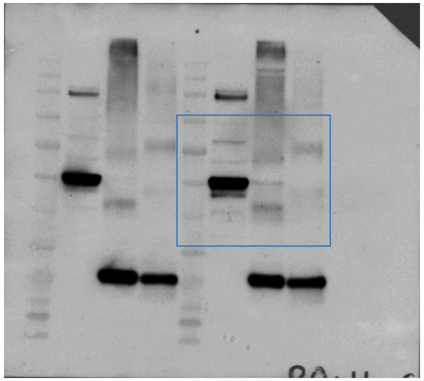

Uncropped original Western blot image for Figure 5A (right)
(The area shown in Figure 5A (right) is indicated by the blue line)

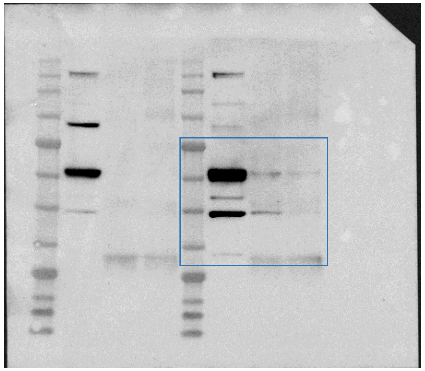

Uncropped original Western blot image for Figure 5B (left)
(The area shown in Figure 5B (left) is indicated by the blue line)

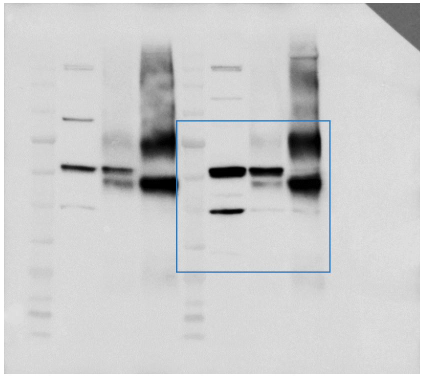

Uncropped original Western blot image for Figure 5B (right)
(The area shown in Figure 5B (right) is indicated by the blue line)

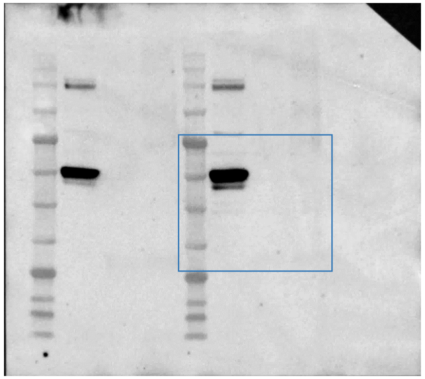

Uncropped original Western blot image for Figure 5C (left)
(The area shown in Figure 5C (left) is indicated by the blue line)

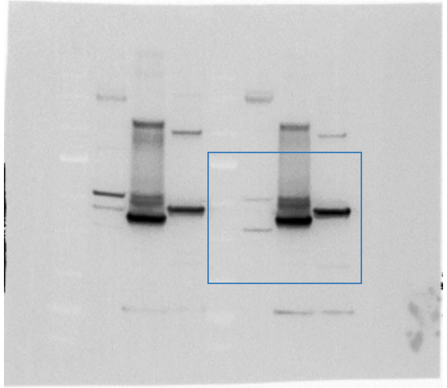

Uncropped original Western blot image for Figure 5C (right)
(The area shown in Figure 5C (right) is indicated by the blue line)

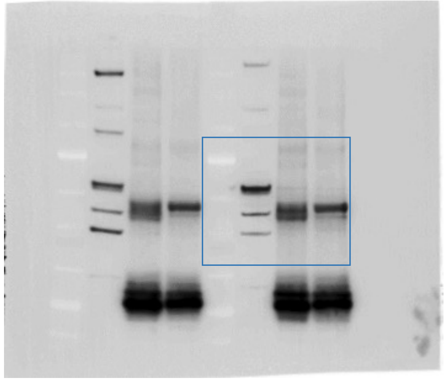

Uncropped original Western blot image for Figure 5D (left)
(The area shown in Figure 5D (left) is indicated by the blue line)

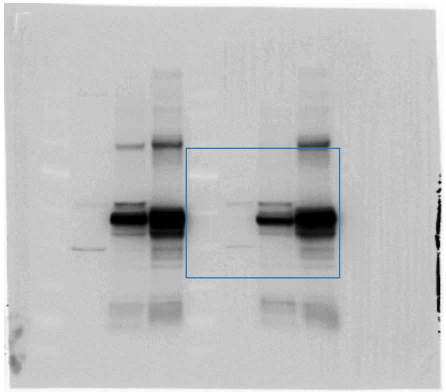

Uncropped original Western blot image for Figure 5D (right)
(The area shown in Figure 5D (right) is indicated by the blue line)

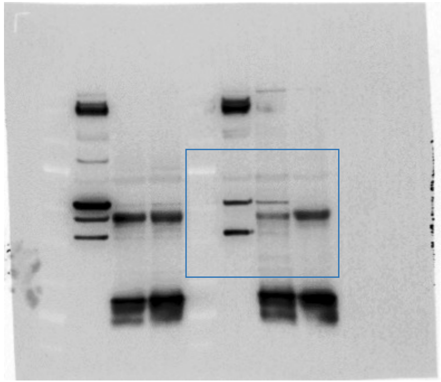

Uncropped original Western blot images for Figure 7A (left)
(The area shown in Figure 7A (left) is indicated by the blue line)

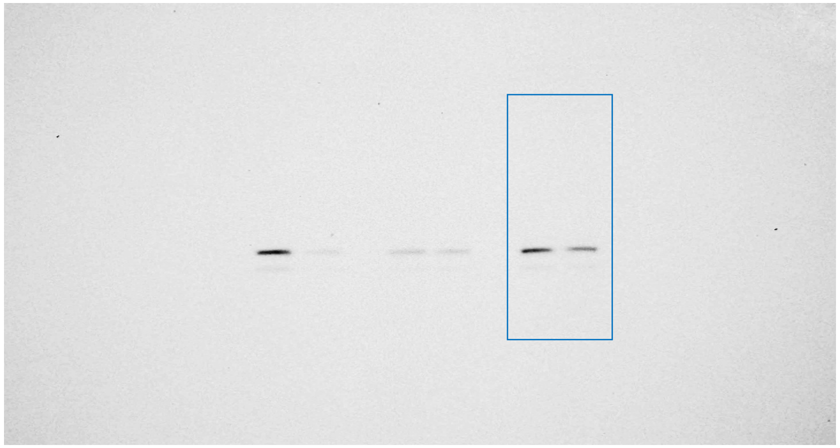

Uncropped original Western blot images for Figure 7A (right)
(The area shown in Figure 7A (right) is indicated by the blue line)

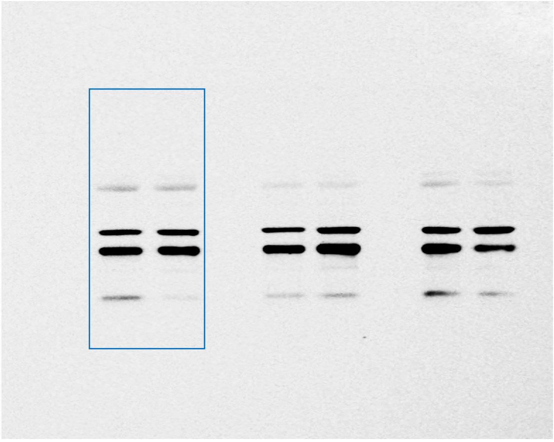

Uncropped original Western blot images for Figure 7B (upper left)
(The area shown in Figure 7B (upperleft) is indicated by the blue line)

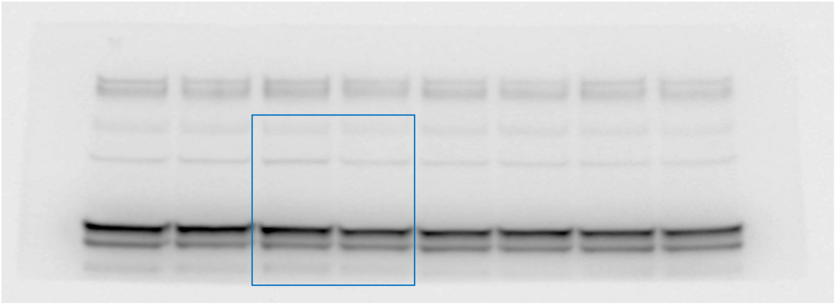

Uncropped original Western blot images for Figure 7B (middle left)
(The area shown in Figure 7B (middle left) is indicated by the blue line)

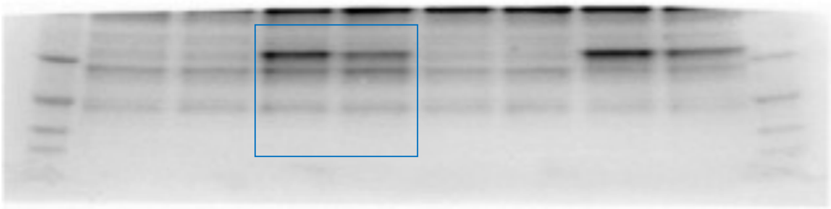

Uncropped original Western blot images for Figure 7B (right)
(The area shown in Figure 7B (right) is indicated by the blue line)

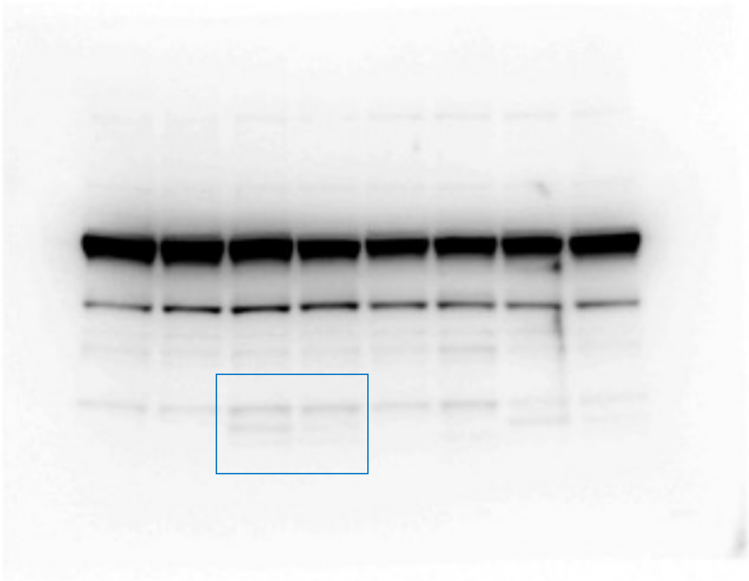

Uncropped original Western blot images for Figure 7C (left)
(The area shown in Figure 7C (left) is indicated by the blue line)

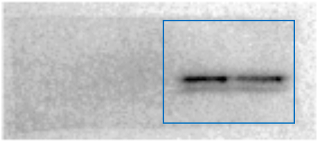

Uncropped original Western blot images for Figure 7C (right)
(The area shown in Figure 7C (right) is indicated by the blue line)

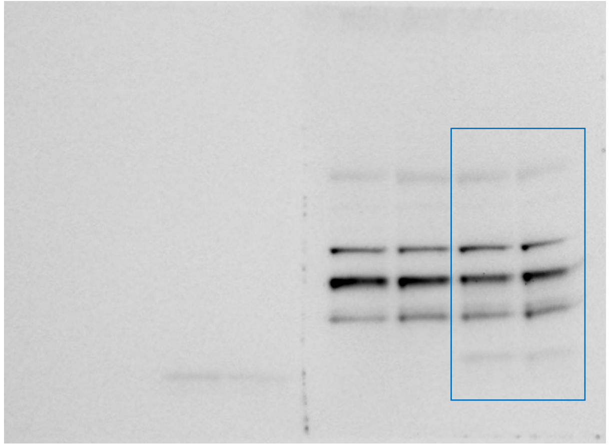

Uncropped original Western blot images for Figure 7D
(The area shown in Figure 7D is indicated by the blue line)

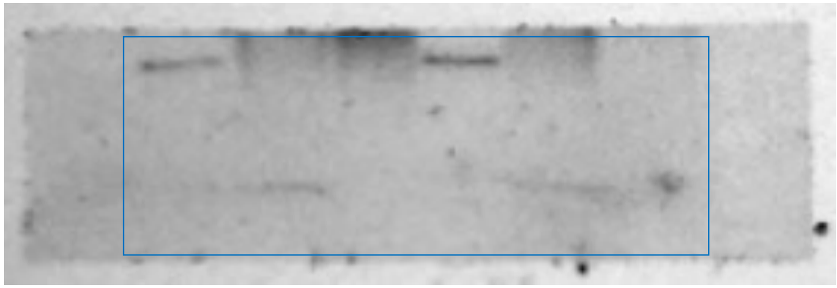

Uncropped original Western blot images for Supplementary Figure S5A
(The area shown in Supplementary Figure S5A is indicated by the blue line)

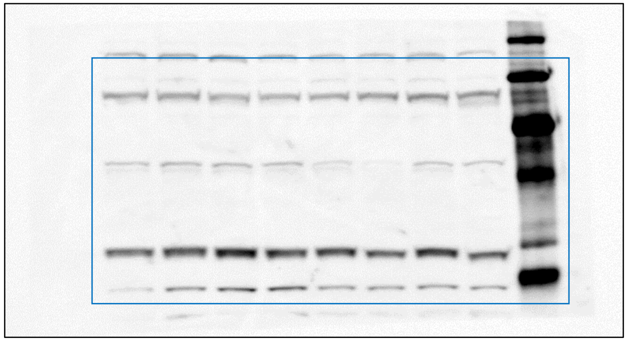

Uncropped original Western blot images for Supplementary Figure S5B

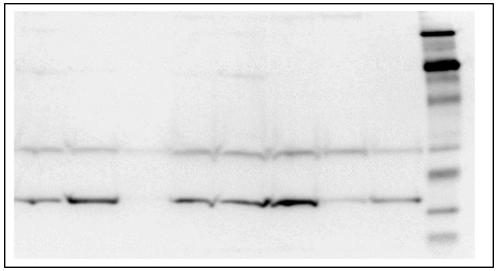

Uncropped original Western blot images for Supplementary Figure S6 (left)
(The area shown in Supplementary Figure S6 (left) is indicated by the blue line)

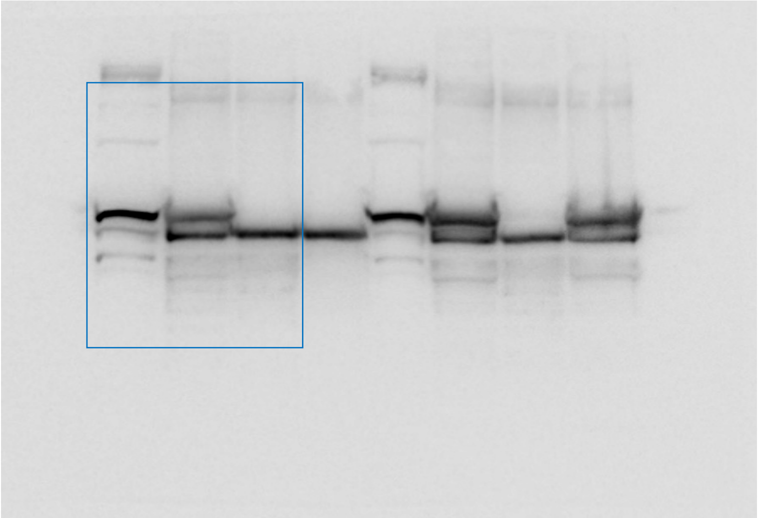

Uncropped original Western blot images for Supplementary Figure S6 (right)
(The area shown in Supplementary Figure S6 (right) is indicated by the blue line)

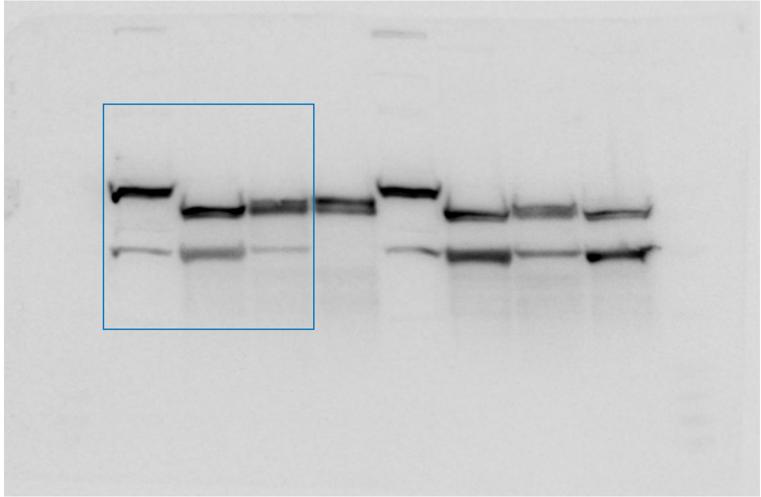

Supplement: Supplementary file 3 — Uncropped original Western blot images [file 41419_2024_6799_MOESM3_ESM.pdf]
